# Sexual Debility in Man

**Published:** 1901-03

**Authors:** 


### Book Reviews.

SEXUAL DEBILITY IN MAN. By FREDERIC R. STURGIS, M. D., formerly Clinical Professor of Venereal Diseases, Medical Department, University of the City of New York; Ex-Visiting Surgeon to the City Hospital, Blackwell's Island; author of "A Manual of Venereal Diseases;" one of the authors of "A System of Legal Medicine," etc. Small 8vo, pp. 432. Illustrated. New York: E. B. Treat & Co. 1900. [Price, $3.00]

This work in contradistinction to others of a similar kind deals only with sexual diseases in the male, the first three chapters being devoted to the physiology of the sexual organs.

The chapter on masturbation should be read carefully by every conscientious physician. In his statement of causes of the nervous phenomena accompanying this condition, Sturgis has failed to mention possibly the most important and to the reviewer the very thing that is liable to bring about derangement of the mental faculties in one who has inherited a weakened, degenerated nervous system. We refer to the quack literature that is today flooding the country, every sentence teeming with suggestions of the madhouse and other horrible endings.

One of the most valuable suggestions as a cure is the advice to use hypnotism, for nothing can be imagined of a easier nature than to produce the hypnotic state in some, if not all, who practise masturbation beyond a certain time. If inquiry is made it is usually found that in patients a slave to this form of amusement, father, mother or grandparents have suffered, or at present are sufferers from some marked degenerated condition of the nervous system, possibly some of the family being confined in a madhouse. Is it folly to suppose that the offspring of such parents would have any other than a weakened nervous system which could be easily swayed to right or wrong, especially toward the latter? The author makes no specific mention of drug treatment for these patients. This is a sad omission, for with these individuals most gratifying results are obtained from medication in the shape of tonics.

Sturgis must be most seriously disagreed with when he infers that onanism, practised even in moderation, is not followed by some bad results, and this in the absence of any inflammatory condition of the parts (sexual apparatus), either simple or specific.

Where treatment is called for, after the stoppage of the habit, it is pleasing to hear the author advise silver in weak solutions, for there is no doubt that this drug is called for in the treatment of pathological conditions of the urethral membrane, due to the practice of onanism; the condition is often prolonged solely by the use of argentum in strong solutions.

Sturgis very pointedly declares there is no connection between nocturnal emissions and spermatorrhea, *i. e.*, although spermatorrhea occasionally follows in the wake of emissions, it does not depend upon the former nocturnal emissions as its starting point. He must be disagreed with most emphatically when he states, "almost all cases of genuine spermatorrhea occur without any antecedent nocturnal or diurnal pollutions." To a certain extent he may be right, but from most careful questioning in a large number of cases of this description, nightly emissions have usually been found to precede the loss of semen without the patient's knowledge—erections, sensation, and the like. The very thing he states, insufficiency of the ejaculatory duct being necessary to true spermatorrhea, is to the reviewer the exact condition that is developed by frequent emissions. A case under treatment now, we are sure, goes a long way to prove this; gonorrhea first and only time two years ago, this followed by frequent nocturnal emissions, which gradually diminished, but as they became less, a whitish substance was seen at the meatus during the day, especially after the bowels moved. Examination proved this to be seminal fluid. As the latter condition became well established, nocturnal pollutions ceased altogether, the other continuing until relieved by treatment. Although this patient is 35 years old, nothing was ever seen at the meatus previous to the commencement of emissions. There was no history of excessive masturbation nor had he ever suffered before from nightly losses. It must not be inferred that the reviewer claims that all cases are a sequence of emissions, for cases are often seen where eliminating the occasional emission of a healthy man, this disease is found developed in marked form. Although the author does not express himself very clearly, we do not believe that spermatorrhea can be considered a disease, *per se*, any more than can be jaundice, vomiting or coughing; but rather that it is only a symptom of some pathological condition. As to the mental disturbances and alienations produced in patients through pollutions, the author cannot be too heartily agreed with in his opinion, that where such take place, the inherited tendencies, etc., must be taken into consideration.

It can safely be asserted that individuals suffering to any extent from mental disturbances due to pollutions, are the offspring of parents who have transmitted to them weakened nervous systems, or weakened mental faculties to an extent that they are easily swayed. When such occurs it is generally the result of the reading of quack literature—a cause which Sturgis should have mentioned.

In considering the treatment of emissions, the reviewer must consider it an unconscious omission on the part of the author, to have failed to mention the steel sound in the care of this trouble. Surely, no one who has had any experience in the treatment of pollutions, either diurnal or nocturnal, can fail to recognise the great benefit received from the sound.

To sexual impotence 191 pages are given up, a large portion of the space being devoted to the citation of cases coming under the heading of that form termed psychical. Most of the histories given appear in works of this or a similar kind and are very interesting reading; not that any knowledge can be gained therefrom, but they go a long way toward proving a statement once made: "everybody is crazy on some one subject." Surely, any one reading the doings of the patients referred to must form but one conclusion, namely, that they are insane; and furthermore they should not be allowed to roam at large, but should be confined until their brains become balanced. Here, as in the treatment of emissions, Sturgis has failed to mention the sound, undoubtedly the most valuable aid in treating that form, the most common variety,—irritative,—due to a hyperesthesia of the posterior urethra. This is a serious oversight, if such it is, for there is no doubt that the sound exerts a more powerful influence on the deep urethral tissues than would a barrel of medicine. Sturgis impresses one with his firm convictions, and it must be said, with a few exceptions, they are good common sense opinions and should receive the credit due them.

The volume should be in the hands of every practitioner of medicine who should carefully read and carry into practice the author's ideas, for only by so doing can any young men be saved who are being treated at the hands of charlatans.—J. H. D.

